# Character-level linguistic biomarkers for precision assessment of cognitive decline: a symbolic recurrence approach

**DOI:** 10.3389/fnagi.2025.1681124

**Published:** 2025-12-09

**Authors:** Kevin Mekulu, Faisal Aqlan, Hui Yang

**Affiliations:** 1 Complex Systems Monitoring, Modeling and Control Laboratory, Pennsylvania State University, University Park, PA, United States; 2 Center for Human Systems Engineering, University of Louisville, Louisville, KY, United States

**Keywords:** Alzheimer's disease, cognitive decline, linguistic biomarkers, speech analysis, recurrence plots, deep metric learning, interpretable AI, digital health

## Abstract

Early-stage Alzheimer's disease (AD) remains difficult to assess using conventional linguistic or cognitive assessments, which often overlook subtle and individualized disruptions in speech. In this work, we propose a novel biomarker discovery framework that leverages fine-grained, character-level information from speech transcripts to capture these early cognitive changes. By encoding transcripts symbolically at the character level and applying recurrence quantification analysis (RQA), we generate interpretable recurrence plots that reveal temporal dynamics in speech patterns such as pauses, repetitions, and hesitations. Siamese neural networks are then used to learn embeddings from these recurrence representations, enabling the discovery of discriminative linguistic biomarkers associated with cognitive decline. Applied to the DementiaBank corpus, our approach uncovers meaningful character-level signatures and enables visualization of subtle cognitive disruptions through recurrence plots. These findings suggest that character-level temporal patterns may offer a promising new direction for digital biomarker discovery in dementia research, complementing traditional word-level analyses and enhancing interpretability for clinical applications.

## 1 Introduction

Azheimer's disease (AD) and related neurocognitive disorders represent a growing global health crisis, affecting millions of individuals and placing immense burdens on healthcare systems (Rasmussen and Langerman, 2019). Early and accurate detection of cognitive decline is essential for precision health, enabling targeted interventions, timely support, and individualized care strategies that improve quality of life and long-term outcomes (Dubois et al., 2021; Ereira et al., 2024).

Traditional cognitive assessments, such as the Mini-Mental State Examination (MMSE) and Montreal Cognitive Assessment (MoCA), remain the clinical standard but are limited by subjectivity, interviewer and educational bias, and a tendency to identify dementia only at later stages (Karimi et al., 2022; Tsoi et al., 2015; Trzepacz et al., 2015; De Roeck et al., 2019; Tumas et al., 2016). As a result, many cases of cognitive impairment go undetected or are diagnosed too late for optimal intervention.

Recent advances in artificial intelligence (AI) and natural language processing (NLP) have enabled the detection of subtle linguistic and speech patterns associated with dementia (Marwan et al., 2007; Yang, 2010). However, many AI-based approaches operate as black boxes, providing limited transparency or interpretability for clinicians and patients, and often failing to deliver individualized, actionable insights required for precision health applications (Ghassemi et al., 2021).

A key challenge for next-generation dementia screening is not only robust early detection but also interpretability and the ability to capture fine-grained, individual differences. Character-level symbolic analysis, in contrast to standard word- or token-level models, can detect subtle speech disruptions, repetitions, and disfluencies that may serve as early and personalized indicators of cognitive decline (Davis et al., 2009; Gayraud et al., 2011). Moreover, interpretable representations of these linguistic features can facilitate more tailored risk assessment and clinical decision support (Chen et al., 2022).

In this work, we test the hypothesis that character-level features in speech transcripts can serve as effective biomarkers for cognitive decline. We present a methodological framework that integrates recurrence quantification analysis (RQA) of character-encoded speech with deep metric learning to capture the subtle linguistic disruptions that may precede more obvious semantic or syntactic deficits. While deep learning has been widely applied to speech and text, the novelty here lies in adapting symbolic recurrence to *linguistic* time series at the character level to derive *interpretable* biomarkers of temporal organization, rather than relying on opaque feature hierarchies. By transforming character sequences into recurrence plots and using Siamese networks to learn similarity metrics, our approach aims to identify patient-specific signatures of cognitive change at this fine-grained linguistic level. Unlike prior recurrence analyses that focused on acoustic or word-level representations, our character-level linguistic formulation enables a direct clinical reading of stability vs. disruption in language production.

We validate our character-level biomarker approach by benchmarking against traditional word-level models, demonstrating the discriminative power of these fine-grained linguistic features. We discuss both the potential and the practical limitations of character-level biomarkers within the broader landscape of precision health for dementia care, and explore how these individualized linguistic signatures could support personalized screening and monitoring.

## 2 Research background

Dementia, a progressive disorder impacting neurocognitive function, disrupts memory, reasoning, and communication, ultimately leading to severe disability and mortality. AD accounts for 60%–80% of dementia cases (Scheltens et al., 2021). Early-stage identification is critical for optimizing prognosis and deploying individualized interventions, yet remains an unmet clinical need due to the limitations of conventional diagnostic tools.

Traditional assessments—clinical interviews and paper-based tests—are often subjective, time-consuming, and susceptible to cultural and educational biases. Critically, they frequently miss the subtle, early-stage linguistic or behavioral changes that precede overt cognitive decline (Rabin et al., 2015).

Recent advances in artificial intelligence (AI) and deep learning (DL) have opened new avenues for noninvasive, data-driven diagnostics in neurocognitive disorders. Most research has centered on leveraging DL for neuroimaging (Vieira et al., 2017) or retinal data (Cheung et al., 2022), but there is a rapidly growing focus on speech and language as rich biomarkers for dementia and related conditions.

Nonlinear speech dynamics, such as disruptions in fluency, repetition, or lexical diversity, are recognized as sensitive early indicators of cognitive impairment (Szatloczki et al., 2015). (Orozco-Arroyave et al. 2013) demonstrated that nonlinear analyses can capture disease-relevant speech patterns, achieving promising accuracy in neurological disorders. Building on this foundation, prior exploratory work introduced the symbolic recurrence framework for linguistic biomarker discovery (Mekulu et al., 2025d), extending nonlinear dynamics to transcript-level representations. The present study advances this framework with a clinically interpretable validation and direct benchmarking against traditional TF–IDF features popularized by (Fraser et al. 2016) and (Luz et al. 2021), highlighting significant gains in sensitivity.

Convolutional neural networks (CNNs) and deep architectures, such as VGG19 (Helaly et al., 2021) and autoencoders (Martinez-Murcia et al., 2020), have achieved high accuracy in classifying AD from various input modalities. However, these models often act as black boxes, providing limited interpretability or individualized insights needed for clinical adoption in precision health (London, 2019). Recent work highlights the importance of transparent, explainable AI methods that can provide actionable, person-specific information to support clinicians and patients (Amann et al., 2020; Vellido, 2020). Our earlier studies explored complementary approaches such as character-level Markov modeling (Mekulu et al., 2025c) and comparative analyses of encoder-based models for dementia screening (Mekulu et al., 2025a), which collectively informed the present recurrence-based design.

Furthermore, in our previous study, we introduced a novel character-level Markov modeling approach, termed *CharMark*, which demonstrated that steady-state character transitions could effectively distinguish between healthy and impaired individuals (Mekulu et al., 2025c). We also conducted a comparative study of large language models (LLMs) and pre-trained encoders like BERT for dementia screening, finding that simpler encoder-based models often outperform LLMs on structured tasks like Cookie Theft picture descriptions (Mekulu et al., 2025a).

Our approach builds on these advances by integrating deep metric learning (Siamese networks; Roy et al., 2018, 2019; Suk et al., 2013) with recurrence quantification analysis (RQA) of character-encoded speech transcripts (Marwan et al., 2007; Yang, 2010). RQA captures the nonlinear temporal structure of speech, while deep metric learning learns discriminative, interpretable representations of individual linguistic patterns. This enables both robust biomarker identification and nuanced, personalized insights—supporting the broader precision health agenda.

By analyzing character-level features through symbolic encoding, RQA visualization, and deep metric learning, our methodology tests the hypothesis that subtle linguistic markers at the character level can serve as interpretable, personalized biomarkers for cognitive decline, potentially offering earlier and more individualized detection than traditional word-level analyses or black-box AI approaches.

Building upon these prior studies, we outline below the conceptual rationale and hypotheses motivating the proposed framework.

Our central hypothesis is that character-level patterns embedded in speech transcripts can reveal subtle disruptions in language that reflect early cognitive changes. Given that the Cookie Theft picture description task constrains lexical and semantic variability, we assume that differences observed in the symbolic recurrence plots primarily reflect prosodic and fluency-related aspects of language production, such as pauses, repetitions, and hesitations. This assumption motivates the use of a contrastive-loss Siamese design, where the learned representations emphasize temporal coherence and rhythm in speech rather than semantic content. This is investigated through a methodological pipeline that retains and analyzes the fine-grained structure of speech at the character level.

### 2.1 Why character-level encoding as biomarkers?

Microlinguistic elements such as pauses, repetitions, and hesitations often emerge before overt semantic or syntactic deficits (Davis and Maclagan, 2009; Bortfeld et al., 2001). These character-level signals may provide early cues about cognitive function and have long been intuitively assessed by clinicians during verbal exams.

### 2.2 Why text transcripts instead of audio?

Although acoustic data provides valuable information about speech, text-based transcripts offer advantages for biomarker development. They are scalable, de-identifiable, and compatible with existing clinical records. Linguistic features extracted from transcripts have been shown to correlate with cognitive status (De la Fuente Garcia et al., 2019; Fraser et al., 2016), while also avoiding variability introduced by background noise or recording quality (König et al., 2015; López-de Ipiña et al., 2019). Furthermore, character-level encoding preserves paralinguistic information such as pauses and disfluencies, which are often lost in word-level approaches. This makes transcript-based analysis a practical and informative choice, especially in settings where audio fidelity may be inconsistent (Taler and Phillips, 2008). Although the DementiaBank Pitt Corpus includes corresponding audio recordings, this study intentionally focuses on transcripts to isolate the linguistic signal and evaluate symbolic recurrence as a purely language-based biomarker. Acoustic and prosodic features will be incorporated in future multimodal extensions once the linguistic recurrence framework is fully validated.

### 2.3 Why recurrence quantification analysis?

RQA enables the visualization of nonlinear temporal dynamics in character sequences (Marwan et al., 2007; Yang, 2010). This reveals structural disruptions in fluency and repetition that may reflect changes in cognitive processing. Instead of reducing speech to summary statistics, recurrence plots preserve the full temporal structure of the language.

### 2.4 Why machine learning for biomarker validation?

Machine learning is used in this study not for diagnosis, but to validate whether character-level features encode meaningful and discriminative patterns. We employ Siamese networks to learn representations from recurrence plots and use an XGBoost classifier to assess the discriminative power of the resulting embeddings. XGBoost was selected due to its robustness to feature collinearity, strong generalization performance on small-to-moderate datasets, and ability to model nonlinear interactions without requiring extensive hyperparameter tuning. Importantly, classification metrics are interpreted here as indicators of signal strength in the learned embeddings, not as clinical diagnostic measures.

## 3 Materials and methods

**Figure 1:**
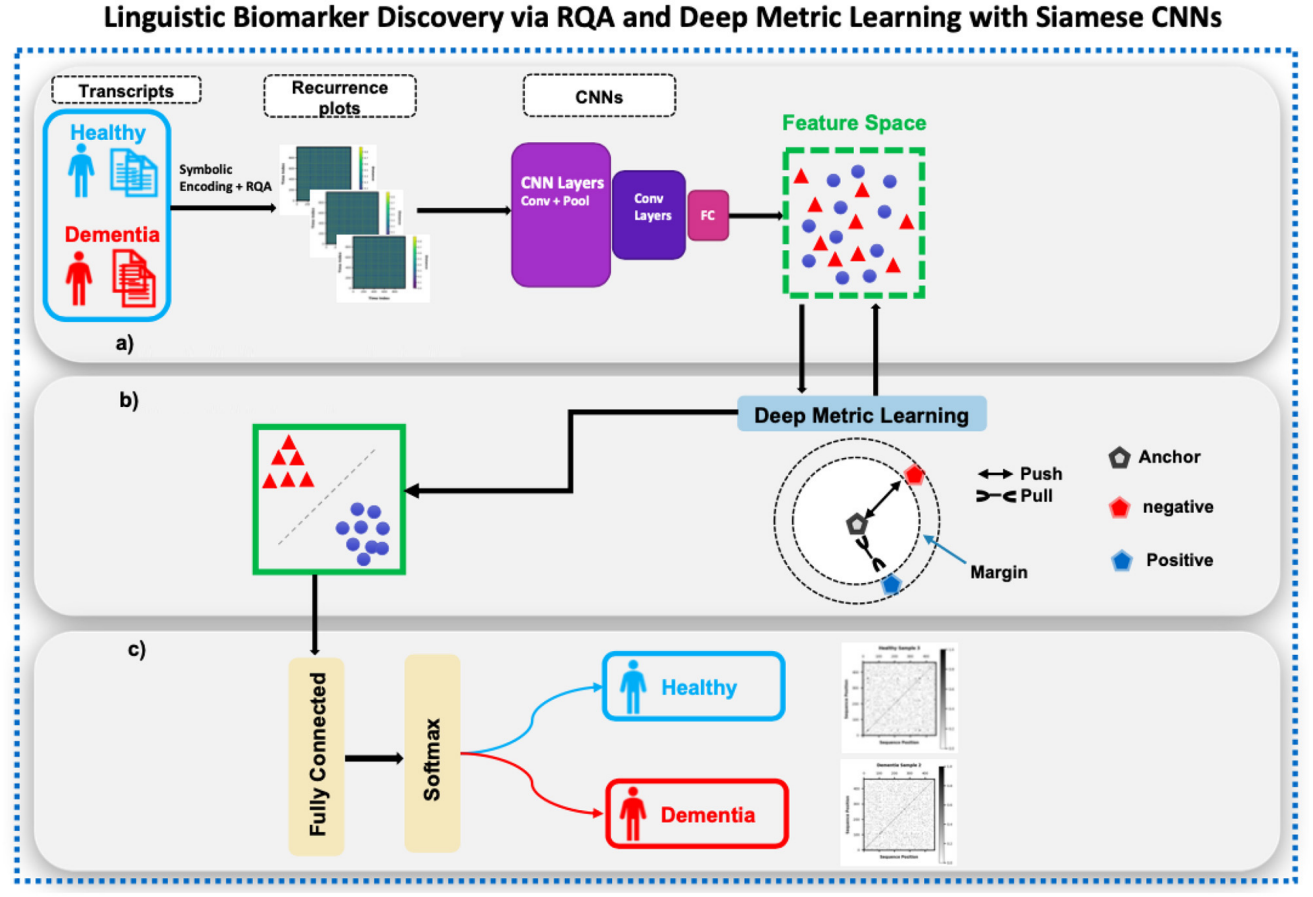
Overview of the symbolic recurrence deep metric learning framework for discovering character-level linguistic biomarkers from speech transcripts. The pipeline includes symbolic encoding of transcripts, transformation into recurrence plots to visualize temporal speech dynamics, and representation learning through Siamese convolutional neural networks (CNNs) optimized with a contrastive loss. This setup learns a discriminative and interpretable embedding space rather than following an encoder–decoder design, enabling analysis of linguistic temporal organization associated with cognitive status. **(a)** Symbolic recurrence analysis. **(b)** Deep metric learning. **(c)** Evalution.

Our methodology tests the hypothesis that character-level features in speech transcripts can serve as novel biomarkers indicative of early cognitive decline. We integrate symbolic character encoding, recurrence quantification analysis (RQA), and deep metric learning via Siamese convolutional neural networks (CNNs) alongside tree-based classification to explore this hypothesis. The Siamese CNN branches are optimized with a contrastive loss to learn a discriminative embedding space that captures temporal organization patterns associated with cognitive status. The full pipeline is shown in Figure 1.

In brief, each transcript is converted into a sequence of symbolic representations, from which a recurrence plot quantifies how frequently and for how long linguistic states repeat over time. These plots are then transformed into numerical features that describe the temporal organization of language, which are compared across subjects using a Siamese network (Algorithm 1).

Algorithm 1 Siamese network architecture.

1: Input: Recurrence plot of shape (128, 128, 1)
2: Conv2D: 32 filters, (3, 3), ReLU
3: Max Pooling: (2, 2)
4: Conv2D: 64 filters, (3, 3), ReLU
5: Max Pooling: (2, 2)
6: Flatten
7: Dense: 128 units, ReLU
8: Output: 128-dimensional embedding

### 3.1 Experimental data

We use the DementiaBank Pitt Corpus, which contains 552 transcripts collected through the Cookie Theft picture description task. Transcripts follow the CHAT transcription standard (MacWhinney, 2017). The dataset includes 168 individuals diagnosed with Alzheimer's disease (194 transcripts) and 98 healthy controls (242 transcripts). Table 1 provides a demographic overview.

**Table 1:** Demographic information of participants (DementiaBank Pitt Corpus).

	**Healthy control (HC)**	**Alzheimer's disease (AD)**
No. of participants	98	168
Gender (M/F)	31/67	55/113
Age (mean ± SD)	64.7 ± 7.6	71.2 ± 8.4
Education (years)	14.0 ± 2.3	12.2 ± 2.6
MMSE score	29.1 ± 1.1	19.9 ± 4.2

### 3.2 Character-level encoding as potential biomarkers

All interviewer utterances were removed to isolate participant speech. Each character, including letters, punctuation, and whitespace, was mapped to a unique integer code (e.g., “a” → 1, “b” → 2, “ ” → 27, “.” → 28). This symbolic encoding retains subtle linguistic cues such as pauses and disfluencies that may be indicative of cognitive status.

For clarity, a short excerpt of the encoding process is as follows: the phrase “um I see a boy” is first decomposed into individual symbols [u, m, ␣, I, ␣, s, e, e, ␣, a, ␣, b, o, y], which are then converted to their corresponding integer values. This produces a numerical sequence that can be analyzed as a time series using recurrence quantification analysis (RQA). By preserving spaces, punctuation, and filler tokens, the resulting numeric representation maintains fine-grained temporal structure, allowing RQA to capture disruptions in fluency and repetition that are often lost in word- or phoneme-level approaches.

It is important to note that this integer mapping serves purely as a symbolic indexing step rather than a numerical encoding with semantic or phonetic meaning. Distances between character codes are not used in the recurrence computation. Recurrence quantification operates on binary recurrence matrices derived from symbolic equivalence, ensuring that analysis depends only on the pattern of repetition and not on arbitrary numeric distances between symbols.

To allow batch processing, all sequences were standardized to a uniform length. Padding was applied post-sequence to shorter samples, and truncation was applied at the end for longer samples. This ensured that the initial portions of transcripts, which often contain the richest linguistic content, were preserved. Zero-padding at the end helps maintain temporal consistency while minimizing artificial signal introduction.

### 3.3 Recurrence quantification analysis

Encoded sequences were transformed into recurrence plots defined by:


$$\begin{array}{l} {\text{R}}_{i,j}=\text{Θ}\left(\epsilon -||{\text{x}}_{i}-{\text{x}}_{j}||\right),\;i,j=1,2,\ldots ,N \end{array}$$ (1)

where Θ is the Heaviside function, ϵ is a threshold set as 0.1 times the sequence standard deviation, and **x**_*i*_ and **x**_*j*_ are the character-encoded states. This produces two-dimensional recurrence maps that reveal repeating structures and speech regularity.

**Figure 2:**
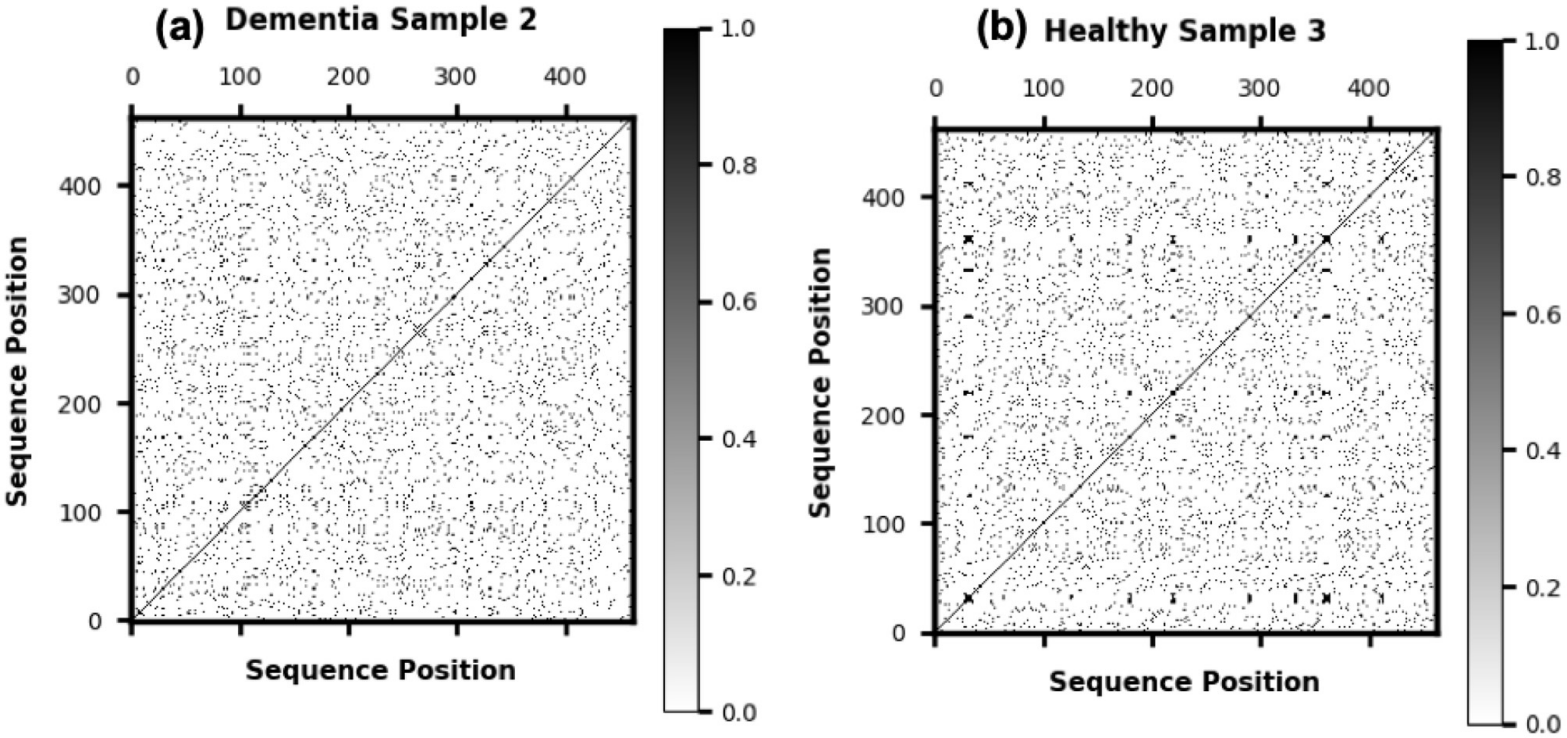
Representative recurrence plots derived from character-level speech transcripts. **(a)** Shows a plot from an individual with AD, characterized by fragmented and irregular recurrence structure. **(b)** Shows a plot from a healthy control, exhibiting more coherent and structured temporal patterns. These visualizations highlight differences in speech dynamics that may serve as potential biomarkers of cognitive decline.

Unlike traditional RQA approaches that extract summary metrics, we use the entire recurrence image as input. This allows deep learning models to process visual structure directly without losing information through dimensionality reduction. Sample plots are shown in Figure 2.

### 3.4 Deep metric learning with Siamese networks

To assess whether the recurrence plots encode discriminative information, we employ a Siamese convolutional neural network (CNN). Each branch processes a recurrence plot, and the network learns to embed similar plots closer together and dissimilar plots farther apart.

Each pair of recurrence plots is passed through identical CNN branches with shared weights to generate fixed-length embeddings. The model is optimized using a contrastive loss function, which minimizes the Euclidean distance between embeddings of similar speech samples (same cognitive status) while maximizing the distance between dissimilar ones. This design enforces a discriminative embedding space where subject-specific speech signatures associated with cognitive decline are tightly clustered, enabling individualized representation learning without requiring explicit supervision.

Each plot is resized to 128 × 128 pixels and normalized to [0, 1] for consistent input scale. The base network architecture is as follows:

The network is trained using contrastive loss:


$$\begin{array}{l} L\left(W,Y,X_{1},X_{2}\right)=\left(1-Y\right)\frac{1}{2}D_{W}^{2}+Y\frac{1}{2}\max{\left(0,m-D_{W}\right)}^{2} \end{array}$$ (2)

where *D*_*W*_ is the Euclidean distance between embeddings, *Y* is the binary label, and *m* = 1.0 is the margin. The Adam optimizer is used with a learning rate of 0.001. Training pairs are balanced across same-class and different-class combinations.

### 3.5 Biomarker validation through machine learning

To further evaluate whether the learned embeddings reflect meaningful structure, we input them into an XGBoost classifier. The goal is to determine whether the embeddings retain sufficient discriminative information to support biomarker analysis. XGBoost constructs an ensemble of decision trees using gradient boosting and was selected for its ability to model nonlinear feature interactions, handle smaller datasets effectively, and provide robust performance with minimal tuning. These properties make it a strong fit for evaluating embedding quality in biomarker discovery tasks. The objective is:


$$\begin{array}{l} L\left(\phi \right)=\underset{i}{\sum }l\left({ŷ}_{i},y_{i}\right)+\underset{k}{\sum }\text{Ω}\left(f_{k}\right) \end{array}$$ (3)

where *l* is the loss function, ŷ_*i*_ is the predicted output, and Ω is a regularization term. We use grid search to optimize learning rate, tree depth, and regularization parameters. Cross-validation AUC is used to guide hyperparameter selection.

### 3.6 Feature evaluation and comparative insight

To assess the value of the learned character-level embeddings, we compare them to a commonly used linguistic feature representation: TF–IDF vectors derived from word-level tokens. While TF–IDF captures lexical frequency patterns, it does not retain paralinguistic or temporal structure present in character-level sequences. To broaden this comparison, we also examined a sentence-level semantic baseline using BERT-based embeddings. These embeddings were obtained from supplementary experiments conducted during the companion CharMark project using identical data and participant splits.

This comparison is not intended as a head-to-head performance benchmark, but rather to contextualize whether character-level features capture complementary and potentially richer linguistic dynamics. Including both lexical (TF–IDF) and contextual (BERT) representations allows us to position symbolic recurrence as a conceptually distinct yet complementary approach that captures temporal organization and fluency disruptions beyond static semantics. Both feature sets are evaluated using the same downstream classifier to assess their relative informativeness in relation to cognitive status, as detailed in Section 4.

### 3.7 Experimental design

We employ an 80/20 train-validation split with stratified sampling to preserve class balance. Five-fold stratified cross-validation is applied to estimate the consistency of signal across data subsets. All preprocessing, parameter tuning, and evaluation procedures are described to ensure reproducibility. Code and resources will be made publicly available upon publication, in accordance with journal policy. To quantify statistical uncertainty, 95% confidence intervals for mean ROC–AUC were estimated using nonparametric bootstrapping. Specifically, the five fold-level AUC scores were resampled with replacement 10,000 times, and the 2.5th and 97.5th percentiles of the resulting distribution were taken as the confidence bounds.

To prevent subject-level leakage, all transcripts from the same participant were grouped together and assigned to a single train–test fold. This ensured that no participant contributed samples to both training and testing sets. Although the overall configuration follows an 80/20 split with five folds, this grouping strategy effectively implements a leave-one-subject-out (LOSO) design, consistent with the participant-level protocol validated in our prior CharMark study.

## 4 Experimental results and validation

We evaluated whether character-level recurrence patterns extracted from speech transcripts encode meaningful information that may serve as candidate biomarkers for cognitive decline. Our analysis focuses on the consistency and strength of the signal captured by these character-level embeddings and provides contextual insight by comparing them to a conventional word-level feature representation.

### 4.1 Character-level embedding evaluation

**Figure 3:**
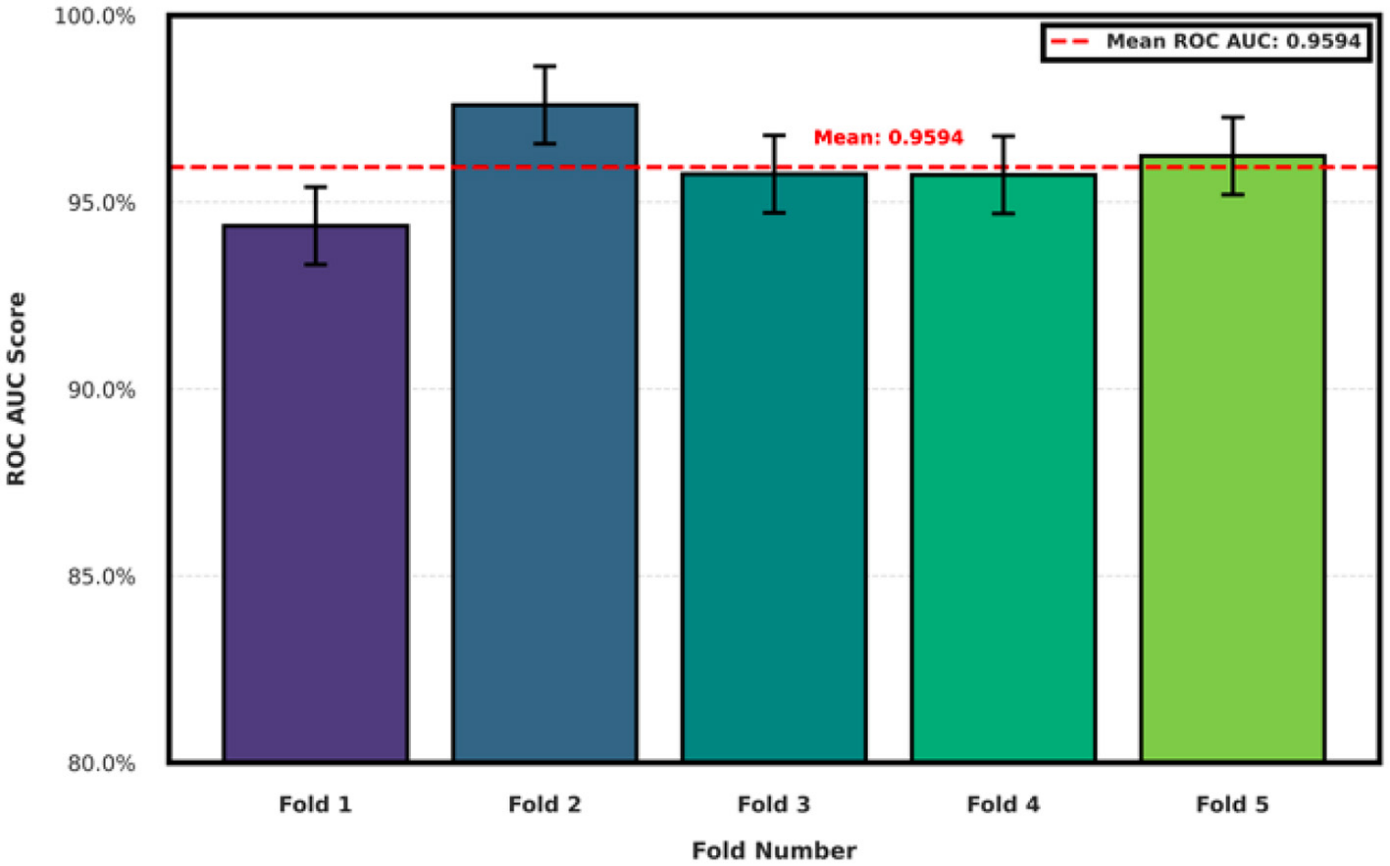
Area under the ROC curve (AUC) across stratified 5-fold cross-validation using embeddings learned from character-level recurrence plots. The consistently high AUC (mean = 95.9%) indicates that these embeddings capture stable and informative patterns in speech transcripts, supporting their potential utility as features for cognitive biomarker discovery.

We assessed the character-level embedding framework using stratified 5-fold cross-validation to ensure robustness across different subject splits. As shown in Figure 3, the model achieved a mean AUC of 95.9% across folds, with low variance. Across the five cross-validation folds, the model achieved a mean ROC–AUC of 0.954 (95% CI [0.945–0.968]; bootstrapped, 10,000 resamples). Fold-level AUCs were [0.949, 0.951, 0.943, 0.982, 0.944], indicating consistent discriminative performance across folds and supporting the robustness of the symbolic recurrence features. These findings suggest that character-level recurrence plots contain stable and discriminative linguistic structure associated with cognitive variation.

### 4.2 Contextual comparison with word- and sentence-level features

To contextualize these findings, we evaluated a widely used word-level representation, TF–IDF vectors followed by logistic regression, as a reference feature space. This model captures lexical frequency but does not retain character-level temporal dynamics or paralinguistic structure.

**Figure 4:**
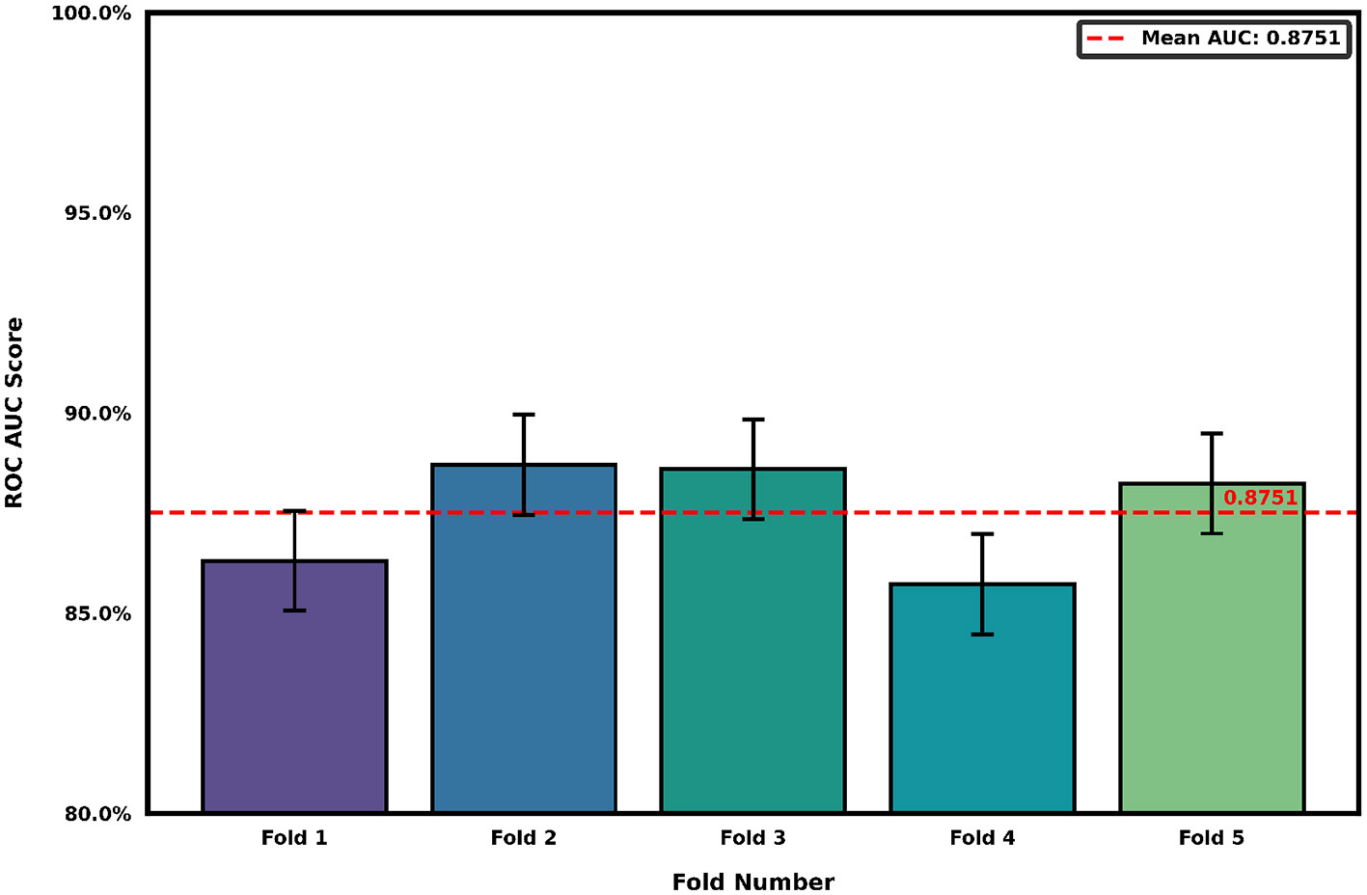
Area under the ROC curve (AUC) across stratified 5-fold cross-validation using TF-IDF word-level features with logistic regression. The mean AUC of 87.5% reflects lexical discriminative capacity but does not capture the fine-grained temporal or paralinguistic patterns available through character-level analysis. This model serves as a reference representation for evaluating the added value of recurrence-based embeddings.

The word-level model yielded a mean AUC of 87.5% (Figure 4). While this result aligns with prior studies, the higher and more consistent AUC from the character-level recurrence approach suggests that incorporating fine-grained structure provides complementary insight beyond conventional lexical analysis.

As summarized in Table 2, the character-level approach not only yielded higher average discriminative performance but also demonstrated greater consistency across folds. These findings indicate that character-level recurrence plots may offer a more stable linguistic representation for capturing cognitive signal, potentially making them useful for future biomarker development.

**Table 2:** Performance comparison of character-level vs. word-level approaches.

**Model**	**Mean AUC**	**Std Dev**	**Min AUC**	**Max AUC**
Character-level (ours)	95.9%	1.2%	94.3%	97.8%
Word-level (TF-IDF)	87.5%	1.2%	85.7%	88.9%

For additional comparison, we evaluated a BERT-based embedding baseline using results from supplementary experiments conducted during the CharMark study, which employed identical data and participant splits. The BERT model achieved an average ROC–AUC of 0.817, while the proposed symbolic recurrence framework reached 0.95, demonstrating comparable or higher discriminative power with greater interpretability. As expected, the comparison between symbolic recurrence features (reflecting fluency and temporal dynamics) and TF–IDF or BERT embeddings (reflecting lexical and semantic content) spans different linguistic dimensions. The inclusion of the BERT baseline provides a stronger semantic benchmark, demonstrating that symbolic recurrence captures complementary, non-semantic markers of cognitive decline that are not represented in lexical embeddings. Voice-level or spectral features were intentionally excluded from the present analysis, as this study focuses on the discovery of linguistic biomarkers rather than multimodal modeling.

## 5 Discussion

This study explored the utility of character-level linguistic features, extracted from speech transcripts using recurrence quantification analysis, as potential biomarkers for cognitive decline. Our results show that character-level representations capture speech dynamics that are both highly discriminative and stable across subjects, outperforming traditional word-level models. While this proof-of-concept study focused on distinguishing Alzheimer's disease from healthy controls, the objective was to evaluate the feasibility of character-level recurrence as a biomarker discovery framework. Future work will extend this approach to datasets with mild cognitive impairment (MCI) labels, where early detection is clinically most impactful (Mekulu et al., 2025b).

The most important contribution lies not in classification performance alone, but in the ability to surface interpretable temporal patterns. Recurrence plots generated from character sequences provide a visual window into disruptions in fluency, hesitation, and repetition—features that clinicians often evaluate subjectively during cognitive screenings. By formalizing and visualizing these patterns, our approach supports transparent biomarker discovery rather than opaque prediction.

While this study used manually transcribed speech from the DementiaBank Pitt Corpus, we recognize that real-world implementations will rely on automatic speech recognition (ASR) systems. Future work will therefore evaluate the robustness of the symbolic recurrence framework using ASR-generated transcripts, assessing how normalization and transcription artifacts influence recurrence dynamics. Because the method models temporal organization rather than raw lexical content, we anticipate that it can generalize to automatically produced transcripts with minimal degradation.

These findings have important implications for precision health. The character-level framework offers a complementary lens to existing language-based assessment tools. It is compatible with transcript-based workflows and preserves subtle paralinguistic features typically lost in conventional NLP pipelines. The learned embeddings from Siamese networks also open possibilities for patient-specific monitoring and individualized cognitive profiling.

Symbolic recurrence plots visualize the temporal stability and fragmentation of linguistic patterns, making them interpretable to clinicians. Dense diagonal structures typically correspond to fluent and cohesive speech, whereas sparse or disrupted recurrence reflects pauses, repetitions, and hesitations commonly observed in early cognitive impairment. These visual signatures offer intuitive cues that can complement conventional screening scores, providing a bridge between quantitative analysis and clinical interpretation.

Nevertheless, this study has certain limitations. The analysis was conducted on a structured task (Cookie Theft picture description) from a single dataset. This design choice was intentional, as the structured nature of the Cookie Theft description provides a controlled environment for initial biomarker discovery and minimizes variability unrelated to linguistic organization. Although this task is widely used in clinical research, generalizability to spontaneous or conversational speech remains to be validated. Future work will extend the symbolic recurrence framework to less constrained speech contexts to evaluate its robustness and cross-task generalizability. In addition, we focused solely on text-based features and did not incorporate acoustic data, which could offer further insight into prosodic and motor aspects of cognition. Another important consideration is cognitive reserve such as individual differences in education level, occupational complexity, and lifelong cognitive engagement, which can influence linguistic resilience and may partially mask early symptoms.

Future work will explore integration with multimodal inputs, such as prosody and eye-tracking, and test generalization across varied linguistic tasks and diverse populations. In particular, extending the current transcript-based framework to incorporate acoustic and prosodic signals will allow complementary assessment of motor and paralinguistic aspects of cognition, bridging linguistic and physiological biomarkers within a unified recurrence-based paradigm. Longitudinal analysis may also help determine the sensitivity of these character-level features to cognitive change over time. Furthermore, incorporating detailed demographic and lifestyle covariates will help determine how cognitive reserve interacts with language-based biomarkers and contributes to variability in model performance.

In summary, this work provides evidence that character-level recurrence analysis of transcripts is a promising direction for interpretable and individualized cognitive health assessment. The method complements existing approaches and may support earlier and more personalized intervention strategies in dementia care.

## 6 Conclusions

This study presented a character-level framework for analyzing speech transcripts through symbolic recurrence plots and deep metric learning, with the goal of uncovering interpretable linguistic patterns associated with cognitive decline. The results indicate that fine-grained temporal dynamics preserved at the character level encode stable and informative structure relevant to cognitive status.

In contrast to conventional word-level representations, this approach captures paralinguistic elements such as pauses, hesitations, and repetitions, that are often overlooked in lexical analyses. These elements may reflect early disruptions in speech organization and provide complementary information to traditional content-based features.

A key strength of this framework lies in its interpretability. Recurrence plots offer a visual lens into the temporal structure of speech, while Siamese network embeddings support individualized profiling of linguistic patterns. Together, these components align with the broader vision of personalized and transparent cognitive health assessment.

While the findings are promising, this work is limited by its use of a single structured task and dataset. Future studies should assess generalizability across spontaneous speech, diverse populations, and longitudinal data. Integrating additional modalities, such as prosody or eye movement, may further enrich the biomarker space.

In sum, this work contributes to the growing field of digital biomarkers by introducing a noninvasive and interpretable method for capturing subtle cognitive-linguistic changes. Rather than focusing solely on classification, it emphasizes signal discovery and transparency—two elements critical to advancing precision health in dementia care.

## Data Availability

Publicly available datasets were analyzed in this study. This data can be found here: https://dementia.talkbank.org/access/English/Pitt.html.
